# Spatial heterogeneity of viral communities across the gastrointestinal tracts of ruminants

**DOI:** 10.1016/j.jare.2026.01.013

**Published:** 2026-01-06

**Authors:** Shizhe Zhang, Qiushuang Li, Yongyi Peng, Jiabin Huo, Tao Ran, Xiumin Zhang, Rong Wang, Jinzhen Jiao, Aoyu Jiang, Guowang Luo, Zhigang Zhang, Qiang Qiu, Zhipeng Li, Shengyong Mao, Zhongtang Yu, Zhiliang Tan, Xiyang Dong, Min Wang

**Affiliations:** a State Key Laboratory of Forage Breeding-by-Design and Utilization, National Engineering Laboratory for Pollution Control and Waste Utilization in Livestock and Poultry Production, Institute of Subtropical Agriculture, Chinese Academy of Sciences, Changsha 410125, China; b University of Chinese Academy of Sciences, Beijing 100049, China; c Key Laboratory of Marine Genetic Resources, Third Institute of Oceanography, Ministry of Natural Resources, Xiamen 361005, China; d College of Pastoral Agriculture Science and Technology, Lanzhou University, Lanzhou 730020, China; e State Key Laboratory for Conservation and Utilization of Bio-Resources in Yunnan, School of Life Sciences, Yunnan University, Kunming 650091, China; f School of Ecology and Environment, Northwestern Polytechnical University, Xi’an 710100, China; g College of Animal Science and Technology, Jilin Agricultural University, Changchun 130118, China; h Laboratory of Gastrointestinal Microbiology, College of Animal Science and Technology, Nanjing Agricultural University, Nanjing 210095, China; i Department of Animal Sciences, The Ohio State University, Columbus, OH 43210, USA

**Keywords:** Viral community, Spatial heterogeneity, Ruminants, Gastrointestinal tract

## Abstract

**Introduction:**

Viruses are abundant biological entities within the gastrointestinal tract (GIT) of ruminants. Current understanding is extensive for bacterial and archaeal communities, but limited for viral communities.

**Objectives:**

The study aimed to investigate viral diversity, virus-host interactions and ecological functions of viruses across GIT regions and ruminant species.

**Methods:**

We collected 373 short-read and long-read metagenomes from 10 GIT regions of seven ruminant species, combining Illumina, PacBio HiFi, and Nanopore sequencing. Viral contigs were identified using sequence homology, viral hallmark gene and machine learning, and employed to uncover community assembly of spatial heterogeneity by analyzing virus-host linkage, lifestyle, and auxiliary metabolic genes (AMGs).

**Results:**

We constructed a Ruminant Gastrointestinal Virome Catalog (RGVC) comprising 43,981 vOTUs, revealing that viral communities were remarkably diverse and mainly driven by the GIT regions rather than by the ruminant species. Virus-host linkage analysis identified 4603 putative prokaryotic hosts across 34 classes for 5954 host-linked viruses, along with robust correlation (*R^2^* = 0.91) observed between abundances of prokaryotic hosts and host-linked viruses across GIT regions. The lysogenic lifestyle was a dominant feature, with integrases being the predominant lysogenic-specific genes. We identified 864 high-confidence AMGs in lysogenic viruses that are annotated as key genes for polysaccharide degradation, glycolysis, and the Wood-Ljungdahl pathway, indicating a putative role for the viruses in supporting these host metabolic functions. The metabolic features of host-linked viruses were further verified by genomic context of selected AMGs of GH10, GPI and FHS with target function.

**Conclusion:**

These findings suggest that the GIT viral communities exhibit spatial heterogeneity with distinct virus-host interactions, and offer new perspectives on maintenance of complex ecological and nutritional functions in ruminant GIT.

## Highlights

• Obtained the Ruminant Gastrointestinal Virome Catalogue (RGVC) with 43,981 vOTUs, revealing expansive viral genomes in ruminants.
• Gastrointestinal regions, rather than ruminant species, are the primary drivers of viral community diversity in ruminants.
• A strong correlation between prokaryotic hosts and host-linked viruses among gastrointestinal regions.
• The prevalence of lysogenic lifestyles can contribute to the observed linkage between viruses and prokaryotic hosts.
• Lysogenic viruses can enhance host metabolism by providing diverse auxiliary metabolic genes.

## Graphical abstract

**Figure:**
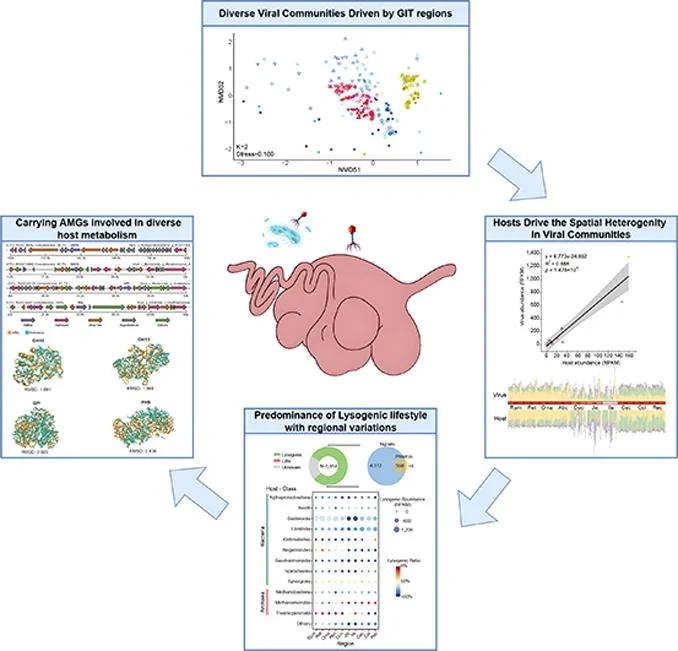

## Introduction

The gastrointestinal tract (GIT) is essential for animal health, playing a pivotal role in nutrient digestion and metabolism [1], [2], [3], [4]. Distinct from monogastric animals, ruminants feature a compartmentalized stomach with four chambers (rumen, reticulum, omasum, and abomasum), which facilitates the digestion of challenging plant cell wall carbohydrates [5,6]. The diverse microbiota within the ruminant GIT, including bacteria, archaea, fungi, protozoa, and viruses, collaborate to facilitate the process of transforming cellulose-rich substrates into volatile fatty acids and microbial biomass for the host [7]. Furthermore, methanogenic archaea in the rumen produce methane by metabolizing substrates such as hydrogen, carbon dioxide, methanol, and methylamine. Among these, hydrogenotrophic methanogens dominate the archaeal community, with *Methanobacterium gottschalkii* and *Methanobacterium ruminantium* as the key representatives [8]. This methane production is a significant contributor to greenhouse gas emissions and global warming [9], [10], [11], [12]. Therefore, the microbial community within the ruminant GIT acts as the central driver, underpinning the host's nutritional physiology while simultaneously presenting significant environmental trade-offs.

Apart from the abundant microbiota mentioned above, viruses are the most abundant biological entities within GIT of ruminants, where they play fundamental roles in shaping microbial community composition, nutrient turnover, and host health. Early studies demonstrated that viral lysis of host cells not only facilitates elemental cycling [13], but also modulates microbial community structure, thereby contributing to the maintenance of homeostasis [14], [15], [16]. Subsequent research has elucidated a more sophisticated metabolic influence, showing that viruses directly reprogram host metabolism either broadly through viral takeover and infection (the ‘virocell’) [17] or more specifically via the expression of auxiliary metabolic genes (AMGs) [18]. Together, these layered roles—from predator to metabolic modulator—underscore the critical ecological importance of viruses within the micro-system [19,20].

Viruses exhibit diverse survival strategies, broadly categorized as lytic or lysogenic lifestyle. Unlike lytic viruses, lysogenic viruses integrate their DNA into the host genome, establishing a symbiotic relationship [21]. In this integrated state, the virus enters a dormant phase as a provirus, which can reactivate under specific conditions to re-enter the lytic cycle [22]. Consequently, lysogenic viruses are uniquely capable of horizontal transmission via the lytic cycle and vertical transmission through lysogeny [23]. However, defining these strategies in real-world contexts has proven extremely complex [22,24]. The selection and transitions of viral lifestyles have considerably impacted their respective hosts. When hosts experience reduced environmental fitness, viruses may enhance lytic activity to promote their own survival, rapidly diminishing host population size [25]. According to the ‘kill-the-winner’ theory, lytic viruses target dominant hosts, thereby promoting the growth of less active microorganisms and fostering diversity in the microbial community [26]. Conversely, the ‘piggyback-the-winner’ theory proposes that lysogeny serves as the primary survival mode for viruses. In this mode, viruses hitch a ride on the reproductive success of their host, adapting to the environment and ensuring their own reproduction [24]. These adaptive strategies also contribute to their host’s survival and ecological fitness as viruses can carry a wide variety of AMGs, expanding and enhancing host metabolism and bolstering host responses to environmental selection pressures [20,27]. The variability of AMGs within viral genomes is influenced by viral lifestyle, habitat, and host species [18], which can contribute to the maintaining of viral diversity within microbiomes.

The advent of high-throughput metagenomics technologies has revealed a vast realm of previously unexplored viral diversity [28], which has significantly advanced our ability to explore the diversity and ecological roles of viruses in the ruminant gastrointestinal tract, as epitomized by the creation of the Global Rumen Virome Database (RVD) [29]. This database, comprising 397,180 species-level viral operational taxonomic units (vOTUs) derived from 975 published rumen metagenomes, represents a significant advancement in our understanding of the rumen virome. Currently, studies have reported that the rumen virome, with its assortment of AMGs, like glycoside hydrolase (GH) 9 and dUTP diphosphatase, can infect core members of the rumen microbiome, such as fiber degraders and methanogens [29], [30], [31], [32]. Additionally, studies have hypothesized that methane emissions could be suppressed by viruses targeting methanogenic archaea, based on the identification of corresponding viral sequences in rumen metagenomes [29,33]. However, research on viral dynamics across various GIT regions of ruminants is still in its nascent stages. Ruminants display a wide range of physiological conditions across different GIT regions, each characterized by distinct functional divisions and significant variations in microbial communities [6]. This complexity is compounded by a viral landscape marked by high diversity, individual variability, and specific distribution patterns within the mammalian gut [34]. This scenario underscores a significant gap in current research, highlighting the pressing need for further studies to delve into the complexities of viral interactions across the different GIT regions of ruminants.

While several studies have investigated rumen viromes [29,32,35,36], comprehensive characterization across the GIT and multiple ruminant species has remained elusive. This knowledge gap limits our understanding of viral diversity, host interactions, and the ecological functions of viruses in ruminant digestion and metabolism. To address this issue, we collected 373 short-read and long-read metagenomes from 10 GIT regions representing seven ruminant species with a total of 6.7 terabase pairs (Tbp) of sequencing data through combined Illumina, PacBio HiFi, and Nanopore platforms. An integrative viral identification pipeline was employed by combining sequence homology, viral hallmark gene detection, and k-mer–based machine learning approaches. We established the Ruminant Gastrointestinal Virome Catalog (RGVC) with nearly 89 % of vOTUs representing novel viruses, marking a substantial expansion of known viral diversity within ruminant GIT ecosystems. Notably, the rumen accounted for the highest viral richness, with distinct heterogeneity of viral diversity and community composition primarily driven by GIT regions. Such spatial variations were closely linked with the different prokaryotic host communities. The revelation of multiple lysogeny-specific genes that foster coexistence and dynamic equilibrium between viruses and prokaryotic hosts also suggests lysogeny as the predominant lifestyle. A diverse set of AMGs, including those involved in the metabolism of carbohydrates and amino acids, further upheld the mutualistic growth and reproduction of both viruses and prokaryotic hosts. These findings provide a comprehensive view of the spatial heterogeneity of viral communities, which can be driven by the interaction of lysogenic viruses with their prokaryotic hosts through AMGs.

## Materials and methods

### Metagenomic data collection and contig assembly

To create a comprehensive analysis of the spatial heterogeneity of gastrointestinal viruses in multi-species ruminants, we collected 370 short-read (second-generation) sequencing metagenomes generated by Illumina technology from 10 GIT regions of seven ruminant species from our previous study, available under accession PRJNA657455 [6]. The 10 GIT regions were stomach (*i.e.,* rumen, reticulum, omasum, abomasum), small intestine (*i.e.,* duodenum, jejunum, ileum), and large intestine (*i.e.,* cecum, colon, rectum), while seven ruminant species were dairy cattle, buffalo, goats, sheep, roe deer, water deer, and yak. We performed *de novo* assembly of the high-quality reads from each sample using two types of software: MEGAHIT v1.1.1 [37] (for efficient large-scale assembly) with parameters: ‘--min-contig-len 500 −t 40′, and IDBA-UD v1.1.3 [38] (for improved handling of uneven sequencing depth) with parameters: ‘--pre_correction --min_contig 500 --num_threads 40 --mink 90 --maxk 124′. The generated contigs were then combined using Minimus2 AMOS v3.1.0 [39] with the parameters: ‘-D CONSERR = 0 OVERLAP = 100 MINID = 100’.

To obtain longer and more complete viral sequences, we collected long-read (third-generation) sequencing metagenomes generated by HiFi and Nanopore technology from our lab. We also obtained HiFi sequencing data from a published study on the ruminant gut microbiome (accession number: PRJNA595610), which was derived from sheep feces and was the only such dataset available at the time of our analysis (2022) [40]. In detail, two rumen microbial samples (one dairy cattle and one goat) were sequenced with HiFi technology, and one dairy cattle rumen microbial sample was sequenced with Nanopore technology. Detailed information about sample collection, DNA extraction, metagenomic sequencing, and quality control is provided in the Supplementary Materials and Methods and Table S1. Contigs were generated by *de novo* assembly using metaFlye v2.8.1-b1676 [41] (‘-pacbio-hifi’ for HiFi and ‘nano-raw’ for Nanopore) and Hifiasm-meta v0.2-r058 [42] (‘-force-preovec’ for HiFi) to generate contigs. For the public sheep fecal microbial dataset, we downloaded the publicly available assembly from Zenodo (https://doi.org/10.5281/zenodo.4729049). Finally, assemblies from the three sources were consolidated, and contigs exceeding 5 kb in length were retained for subsequent viral identification, according to Pratama et al. [43].

### Identification of viral contigs and construction of RGVC

Putative viral contigs were identified through a combination of five methods: (1) contigs were aligned to the IMG/VR v4 database [44] using BLASTn v2.11.0+ (-max_target_seqs 5 -evalue 1e-5 -outfmt 6), retaining hits with ≥90 % identity and ≥75 % coverage; (2) VirFinder v1.1 [45] with a score >0.9 and *p* < 0.05; (3) DeepVirFinder v1.0 [46] with a score >0.9 and *p* < 0.05; (4) VirSorter2 v2.2.3 [47] with a score ≥0.5 with the parameters: ‘-keep-original-seq -seqname-suffix-off’; and (5) VIBRANT v1.2.1 [48] with default parameters. This multi-method strategy ensured robust viral identification by combining: known virus matching (BLASTn vs. IMG/VR), machine learning-based classification (DeepVirFinder, VirFinder), and viral protein detection (VirSorter2, VIBRANT). A total of 1.16 million putative viral contigs were identified by meeting at least one of the filtering criteria.

Subsequently, the obtained putative viral contigs were assessed for quality and trimmed off host sequences using CheckV v1.0.1 [49]. To meet the standards set by the Minimum Information about an Uncultivated Virus Genome (MIUViG) guideline [50], only the contigs exceeding 50 % completeness were retained, resulting in obtaining 85,521 viral contigs. These viral contigs were then compiled and clustered into species-level viral operational taxonomic units (vOTUs) according to 95 % average nucleotide identity (ANI) and 85 % aligned fraction threshold (AF), as implemented in a custom CheckV-derived script [49]. This generated an RGVC comprising 43,981 vOTUs for further analysis.

### Viral taxonomic assignments, clustering and abundance analysis

Taxonomic classification of the vOTUs in the RGVC was performed using geNomad v1.3.3 [51], with reference to the viral taxonomy defined in the International Committee on Classification of Viruses (ICTV)’s Virus Metadata Resource (VMR) (https://ictv.global/). Simultaneously, representative viral genomes from each vOTU were further clustered into viral clusters (VCs) using vConTACT2 v0.11.3 [52] with RefSeq (v85) as the reference database, with parameters: ‘-pcs-mode MCL -vcs-mode ClusterONE -rel-mode Diamond’. The VCs were equivalent to genera of prokaryotic viruses designated by the ICTV. The gene-sharing network of the VCs was visualized in Gephi v0.10.1 [53], following the published protocol in protocols.io (https://www.protocols.io/view/applying-vcontact-to-viralsequences-and-visualizi-x5xfq7n). The abundance of 43,981 vOTUs within 370 Illumina sequencing metagenomes was assessed by mapping against the RGVC using CoverM v0.6.1 [54], with parameters: ‘contig mode; --min-read-percent-identity 95 --min-read-aligned-percent 90 --contig-end-exclusion 0’ [25]. The abundance was expressed in terms of reads per kilobases per million mapped reads (RPKM) to reduce the effects of gene length and sequencing depth on abundances [55,56].

### Viral lifestyle prediction

A provirus represents the latent form of a virus, wherein its genomic material integrates into the host chromosome or persists extrachromosomally, replicating passively alongside the host cell [21,22]. Proviruses were identified from all vOTUs using CheckV through detecting host-genome integration and trimming of host boundary sequences, whereas the remaining vOTUs were classified as free viruses [57].

Viruses exhibit two distinct lifestyles: lysogenic and lytic [58]. Lysogenic viruses are characterized by their ability to undergo the lysogenic cycle as a provirus, which is made possible by lysogeny-specific genes that facilitate the excision and integration processes necessary for genome insertion [23]. On this basis, the vOTUs were assigned as lysogenic lifestyle based on the following two criteria: (1) the genome directly identified as a provirus through host-genome boundary detection by CheckV, which has been explained previously; (2) the genome annotated using VIBRANT (parameter: -virome, via comparison against its built-in viral gene database) showed at least one lysogeny-specific genes (*e.g.*, integrase, recombinase, transposase, resolvase, and excisionase) [18]. For vOTUs that showed no lysogenic signature, those with genomes confirmed by CheckV to be 100 % complete and with a topology characterized by either direct (DTR) or inverted terminal repeats (ITR) were confidently assigned a ‘lytic’ lifestyle [50]. The other vOTUs were categorized as ‘unknown’, as missing regions might contain undetected lysogeny-specific gene.

### Comparisons to viral sequences with public databases

The vOTUs in RGVC were compared to available viral contigs in public databases: (1) The Global Ocean Viromes 2.0 (GOV 2.0, n = 195,728) [59]; (2) the Phages and Integrated Genomes Encapsidated Or Not (PIGEON, n = 266,125) [60]; (3) the Gut Phage Database (GPD, n = 142,809) [3]; (4) IMG/VR v4 (n = 15,677,623) [44]; (5) rumen virome database (RVD, n = 397,180) [29]. The comparison was performed using BLASTn with stringent parameters: ‘-max_target_seqs 5 -evalue 1e-5 -outfmt 6’. Viral genomes shared between the RGVC and other databases were identified based on ≥90 % sequence identity and ≥75 % coverage [3], while the remaining vOTUs in RGVC were classified as novel.

### Host-virus prediction

To predict the probable hosts of the rumen viruses, we manually curated three genome databases according to the method from Yan et al. [29]: a database of 10,373 MAGs assembled from 370 GIT short-sequencing samples in our previous study [6]; a database of 659 qualified MAGs newly assembled from our three long-read sequencing samples (HiFi and Nanopore, binning details are provided in the Supplementary Materials and Methods); and the well-known rumen superset of 5845 publicly available bacterial and archaeal MAG generated at 2019 [61]. The MAGs were taxonomically classified using the GTDB Toolkit (GTDB-Tk) v2.1.1 [62].

Three methods were then employed to predict virus-host linkages according to the literature [27,28,63]. For the first method of CRISPR arrays: CRISPR spacer excising from CRISPR arrays identified in MAGs using MinCED v0.4.2 [64] (default parameters). Spacers shorter than 25 bp and arrays with fewer than three spacers were excluded, whereas spacers were then aligned against vOTUs using BLASTn, requiring 100 % coverage and identity. For the second method of tRNA matching: tRNA sequences were annotated in both vOTUs and MAGs using tRNAscan-SE v2.0.9 [65] with parameters ‘-B’ (for bacteria) and ‘-A’ (for archaea), respectively. Perfect matches (100 % coverage and identity) identified via BLASTn were considered potential linkages. For the third method of nucleotide sequence homology: nucleotide sequence homology with vOTUs and MAGs compared using BLASTn, applying stringent criteria (≥75 % coverage, ≥70 % identity, ≥50 bit score, and e-value ≤0.001) [66]. Hosts predicted by three methods against three genome databases were combined into the database of prokaryotic hosts MAGs. This generated a total of 5954 putative host-linked viruses and 4647 putative host MAGs for further analysis.

### Phylogenetic, functional and abundance analysis of prokaryotic hosts MAGs

A total of 4647 putative host MAGs were firstly dereplicated at 99 % ANI using dRep v3.4.3 [67] (parameters: --ignoreGenomeQuality -pa 0.95 -sa 0.99 -cm larger), yielding 4603 non-redundant host MAGs for downstream analysis. The above prokaryotic host MAGs were then taxonomically classified using GTDB-tk [62] (default parameters) based on the Genome Taxonomy Database (GTDB; version R207_v2). The marker genes from host MAGs were extracted and aligned by following the identification and alignment steps of GTDB-Tk. Phylogenetic trees were constructed using the aligned marker genes and IQ-TREE v2.2.0.3 [68] (parameters: -m MFP -nt 16 -bb 1000 -redo -mredo), applying ultrafast bootstrap approximation (1000 replicates) for branch support, and visualized in iTOL (https://itol.embl.de) [69].

For function annotation, open reading frames (ORFs) of host MAGs were firstly predicted by Prodigal v2.6.3 [70] and annotated using KofamScan v1.3.0 [71] against the KEGG database [72] and using run_dbCAN v4.0.0 [73] against the CAZyme database [74] with default parameters, respectively. The analysis of the abundance of host MAGs in 370 metagenomes sequenced with Illumina was conducted by mapping 4603 non-redundant host MAGs using CoverM [47], with parameters: ‘genome mode; --min-read-percent-identity 95 --min-read-aligned-percent 90 --contig-end-exclusion 0′ [26]. The abundance of host MAGs in metagenomes was also expressed in terms of RPKM by considering both gene length and sequencing depth according to the literature of Zhou et al. [56].

### Functional profiling of metabolic potential in host-linked viruses

To analyze the metabolic potential of host-linked virus, we adopted a conservative approach to confirm and trim sequences of lysogenic vOTUs with known hosts using geNomad [51]. The retained 4738 viral genomes were then processed with VirSorter2 [47] (parameter: --prep-for-dramv) to produce the affi-contigs.tab file and then annotated via DRAM v1.4.6 (viral mode, default parameters) [75]. Following established criteria [43], we classified genes as putative AMGs if they met the following conditions: (1) auxiliary score ≤3; (2) presence of the M marker; (3) absence of A, V, and T markers. Candidate AMGs were further manually validated to ensure that: (1) no ribosomal proteins were present in either flanking region; (2) at least one end contained viral structural genes. Genome linear maps of viral genomes containing AMGs were visualized using Easyfig v.2.2.0 [76] based on DRAM-v annotations. Given that the majority of AMGs originate from the host, we utilized the abundances of vOTUs to reflect the abundances of their AMGs to avoid overestimation from host-derived reads [18].

### Protein structural analysis of AMGs

To determine the three-dimensional structures of the selected AMGs, we performed de novo structure prediction using AlphaFold3 (https://www.alphafoldserver.com) [77]. The predicted models were then structurally aligned against the Protein Data Bank (PDB; https://www.rcsb.org/) to identify the closest matching reference structures for each. The four identified reference structures were 4PMX (for GH10), 5GR5 (for GH13), 3IFS (for GPI), and 5A5G (for fhs). The structural alignment, co-visualization, and the root-mean-square deviation (RMSD) calculation for each predicted AMG and its reference structure were performed in Schrödinger Maestro v14.0, using the Protein Structure Alignment workflow with default parameters.

### Statistical analyses

All statistical analyses were performed using R 4.2.0 with a significance threshold of *p* < 0.05 unless otherwise stated. We assessed viral community structure through Non-metric Multidimensional Scaling (NMDS) ordination based on Bray-Curtis dissimilarity of vOTU relative abundances, comparing both among GIT regions within each ruminant species and across ruminant species for each GIT region. The Bray-Curtis dissimilarity was used as input for PERMANOVA analysis, where ruminant species and GIT regions were used as main effects (adonis function of the vegan v2.6-4 package with 999 permutations) [78]. The vegan package was also employed to calculate α-diversity metrics (Shannon diversity index and Chao1 richness estimate) and perform variation partitioning analysis (‘varpart’) for quantifying community heterogeneity. The correlation between host-linked viruses and host MAGs’ relative abundances in each GIT region was calculated using Pearson’s algorithm.

## Results and discussion

### The ruminant gastrointestinal virome Catalog (RGVC)

To provide a comprehensive overview of the viromes throughout the GIT of ruminants, we collected 373 metagenomic samples from 10 distinct regions of seven ruminant species. We generated a total of 6.7 terabase pairs (Tbp) of sequences using both short- and long-read sequencing techniques, which included 6.5 Tbp from Illumina sequencing (averaging 17.6 Gbp per sample), 77.6 Gbp from HiFi sequencing (specifically from rumen samples of dairy cattle and goats), and 194 Gbp from Nanopore sequencing (from a rumen sample of dairy cattle) (Fig. 1A). Following assembly, the contigs generated in this study were combined with those from Bickhart et al. [40], yielding a combined set of approximately 7.06 million contigs (length ≥5 kb) for downstream viral identification.

**Fig. 1:**
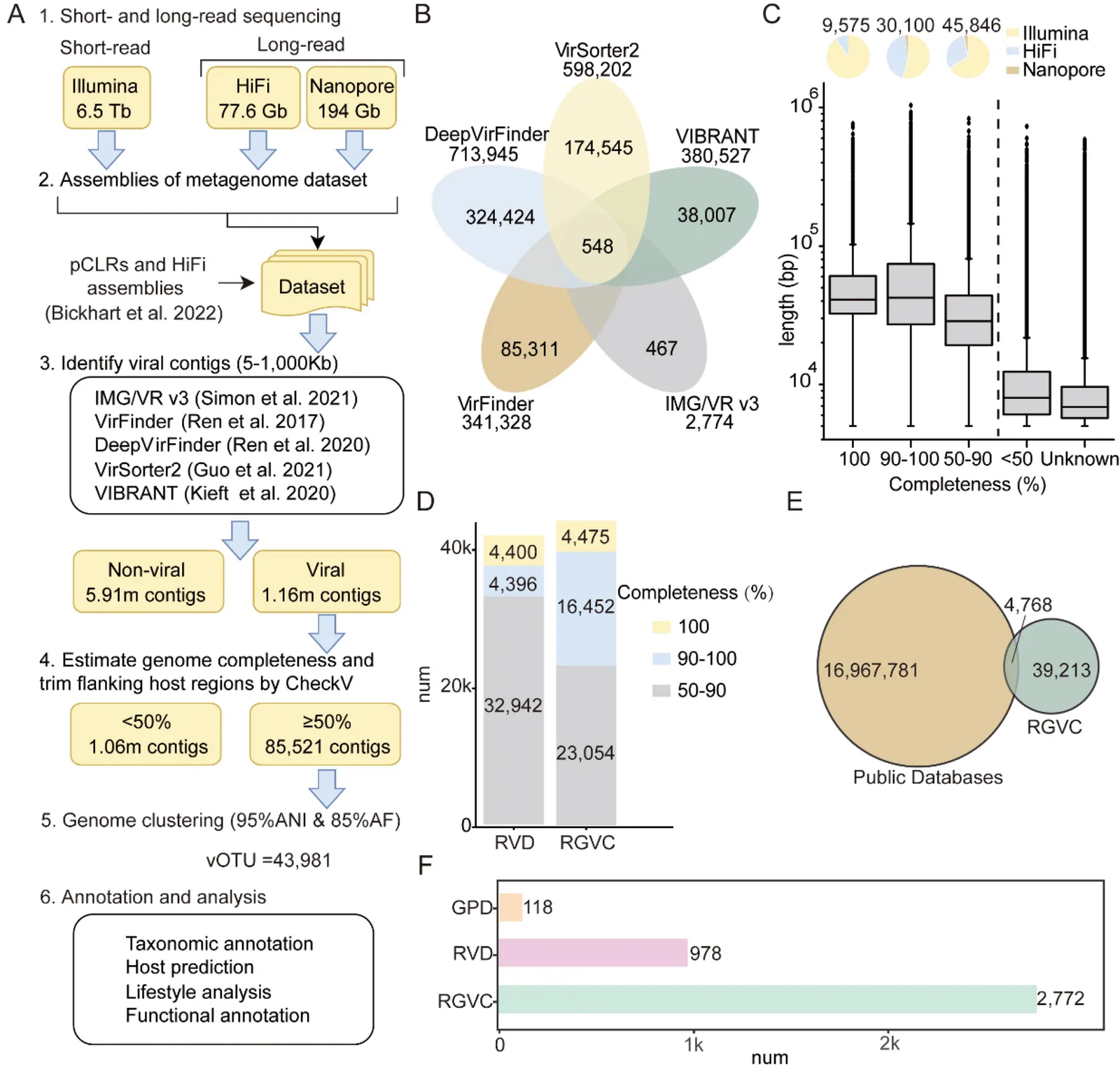
43,981 vOTUs recovered from metagenomes using short- and long-read sequencing. A, Overview of virus discovery efforts and vOTUs formation; B, Diagram depicting the numbers of viruses identified by five different methods; C, Distribution of estimated completeness of vOTUs obtained from short- and long-read sequencing; D, Comparison of the numbers of vOTUs above medium quality in RGVC and RVD, categorized by completeness levels (50–90 %, >90 %, and 100 %); E, Venn diagram displaying the numbers of unique and shared vOTUs between RGVC and public databases, including IMG/VR v4, RVD, GOV 2.0, PIGEON and GPD; F, Numbers of vOTUs with genomes greater than 200 kb in RGVC, RVD and GPD.

Viral identification was performed using five complementary approaches based on three distinct principles, to balance the sensitivity and predictive confidence. First, sequence homology search against the IMG/VR database [44] identified 2774 high-confidence viral contigs with significant similarity to known viruses (Fig. 1B; Table S2). Second, the detection of viral hallmark genes using VirSorter2 [47] and VIBRANT [48] resulted in 598,202 and 380,527 putative viral contigs, respectively. Finally, to maximize the recovery of viral contigs, we incorporated k-mer based machine learning approaches using VirFinder [45] and DeepVirFinder [46], which identified 341,328 and 713,945 putative viral contigs, respectively. The integration of these complementary methods yielded a comprehensive dataset of approximately 1.16 million putative viral contigs. Of these, 61.7 % were detected by DeepVirFinder, primarily from Illumina data (91.2 % of its hits), and 28.0 % were exclusively identified by this tool (Fig. S1). This method appears to exhibit high sensitivity towards short sequence fragments, with relevant reports supporting this conclusion [46]. Then we obtained 85,521 viral genomes with ≥50 % completeness after quality filtering of the 1.16 million putative viral contigs, which represented 7.3 % of the initial dataset (Fig. 1C; Table S3). Further analysis revealed that while the IMG/VR homology-based approach had low recall, it achieved exceptionally high confidence, with a remarkable 99.7 % pass rate in quality assessment for its Illumina-derived contigs. In contrast, DeepVirFinder consistently yielded the lowest pass rate across all three sequencing platforms, validating that its high sensitivity was achieved at the expense of increased propensity for false positives [43]. Collectively, we obtained the RGVC of 43,981 vOTUs (Table S4), based on the Minimum Information about an Uncultivated Virus Genome (MIUViG) guideline: 95 % average nucleotide identity (ANI) and 85 % aligned fraction (AF) [50].

These 43,981 vOTUs in the RGVC were derived from multiple ruminant species, primarily dairy cattle (37.0 %), sheep (21.7 %), and yaks (11.6 %) (Fig. S2A). Notably, the rumen contributed the majority of vOTUs (42.4 % of the total), far exceeding the yields from other GIT regions (*e.g.*, 12.0 % from feces, 9.4 % from duodenum) (Fig. S2B), consistent with previous studies identifying the rumen as the richest reservoir of viral diversity in ruminants [79]. These vOTUs in the RGVC, which included 20,927 non-redundant, high-quality genomes (with completeness ≥90 %), with 4475 of the totals classified as complete (Table S5), which represents a substantial leap forward in the characterization of the virome within the ruminant GIT. This exceeds the number in the RVD [29], which has 8796 high-quality genomes, with 4400 being complete (Fig. 1D). Within this catalog, proviruses, identified as viral sequences flanked by host genomic regions, constituted only approximately 7.01 % of the total vOTUs, while ubiquitously distributed across all ruminant species and GIT regions (Table S6). In contrast, free viruses represented the dominant form in the ruminant GIT, align with observations reported in the human gut [80] and other mammalian systems [34]. The RGVC presents a comprehensive landscape of the ruminant gastrointestinal virome, integrating multi-species, pan-gastrointestinal coverage with rigorous genome quality filtering and precise resolution of viral states, thereby establishing a foundational resource for viral community analysis.

The comparative analysis of the 43,981 vOTUs with publicly available databases such as IMG/VR [44], RVD, and other environmental databases (*e.g.*, GOV 2.0 [59], PIGEON [60], and GPD [3]), reveals that approximately 89.3 % of these vOTUs represent new discoveries (Fig. 1E; Table S7). Moreover, the RGVC showcases a wide range of genome lengths, spanning from 5 kilobases (kb) to nearly 1000 kb. Notably, 1564 viral genomes had lengths less than 10 kb (128 of these were complete), while 2772 genomes had lengths larger than 200 kb (Table S5). The number of large genomes exceeds that of the RVD and the GPD [3], which represents the largest collection of putative phage sequences predicted from the human gut (Fig. 1F). As previously suggested, these large genomes might represent nucleocytoplasmic large DNA viruses (NCLDV) [81] or huge phages [82,83]. Interestingly, these were primarily assembled from the long sequencing reads of Nanopore and HiFi (Fig. S3), indicating the crucial role of long-read technologies in recovering such large viral genomes. The recovery of numerous novel vOTUs and large-genome vOTUs demonstrates the comprehensiveness of the RGVC, thereby providing a solid foundation for characterizing the ruminant viral community.

### Ruminant viral communities are diverse and primarily driven by GIT regions

According to our analytical workflow (Fig. S4), the taxonomic profiling of vOTUs revealed that the majority (n = 42,795) belonged to the class *Caudoviricetes* (Fig. 2A; Table S8). A total of 1019 vOTUs could be classified at the order level, with 489 vOTUs being to the order *Crassvirales*, also known as crAss-like phages, which are recognized as the most abundant viruses in the human gut [84,85]. We also identified 12 vOTUs from the NCLDV, containing members of the order *Imitervirales*, which were affiliated with the class *Megaviricetes* capable of infecting protozoa [86,87].

**Fig. 2:**
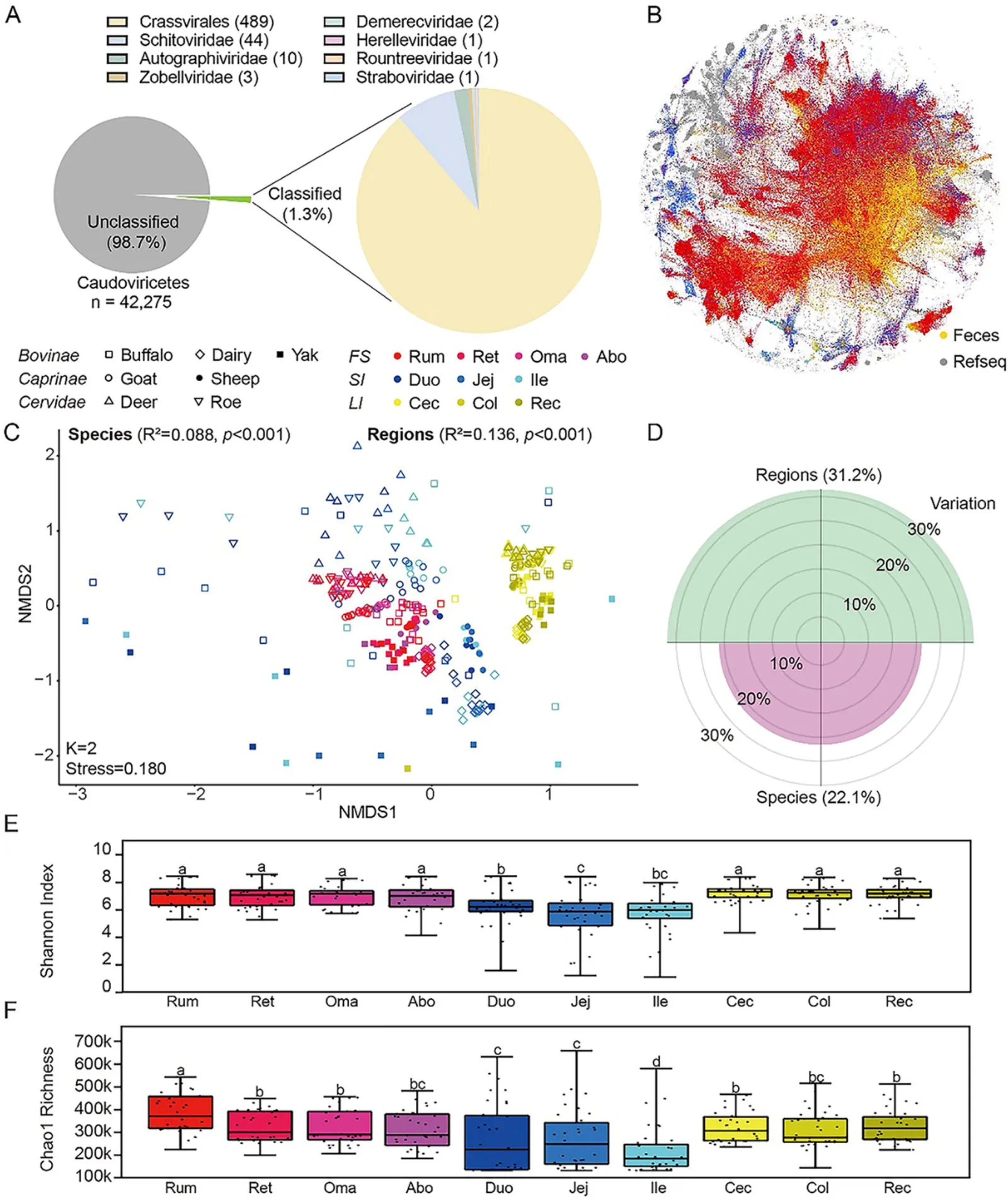
The distinct viral diversity among 10 gastrointestinal regions and 7 ruminant species. A, Taxonomic proportions within the class *Caudoviricetes*. B, Gene-sharing networks of viral sequences in ruminants, based on assemblies from various gastrointestinal regions. Nodes represent viral genomes, and edges indicate similarity based on shared protein clusters. The layout algorithm positions nodes so that their proximity reflects their evolutionary relatedness, which is determined by the number of shared genes. C, Nonmetric multidimensional scaling (NMDS) plot based on the relative abundances (RPKM) of vOTUs. The colors and shapes of the symbols indicate the regions and species, respectively. Bray-Curtis distances associated with regions and species are shown. PERMANOVA was applied to test the difference in viral communities among regions and species; D, Variability in difference explained by regions and species; E, Shannon index for viral community among gastrointestinal regions; F, Chao1 richness estimate for viral community among gastrointestinal regions. Significant differences between means (Duncan’s multiple comparisons, *p* < 0.05) are denoted with different letters. Rum, rumen; Ret, reticulum; Oma, omasum; Abo, abomasum; Duo, duodenum; Jej, jejunum; Ile, ileum; Cec, cecum; Col, colon; Rec, rectum.

According to the criteria established by the ICTV (https://ictv.global/), all the vOTUs were clustered to a total of 3764 VCs (approximately the genus level) (Table S9). These VCs varied widely in the number of vOTU, ranging from 2 to 562. Some VCs exclusively contain vOTUs from a single sample (*e.g.*, VC3021) or an animal (*e.g.*, VC2075) (Table S9), highlighting the uniqueness of viruses for each GIT region and ruminant species. To investigate the clustering pattern of vOTUs across different GIT regions, we employed vConTACT2 to construct a gene-sharing network, based on shared gene clusters between viral genomes [52]. The network showed that few vOTUs (approximately 12.5 %) clustered with taxonomically known genomes from Viral RefSeq [88], highlights the novelty and uniqueness of the ruminant virome we uncovered, suggesting the presence of many previously uncharacterized viral genera (Fig. 2B). Moreover, the vOTUs derived from the stomach were clustered with those from the large intestines, whereas vOTUs from the small intestines were distributed on the periphery. This clustering pattern indicates that the viral communities are highly diverse among the ruminant GIT.

We delved deeper into spatial distribution of viral communities within the GIT and discovered that the abundance of individual vOTUs varied greatly, with averages ranging from 0 to 274 reads per RPKM (Table S10). Only 2797 vOTUs (6.36 % of the total) were present in over 80 % of the samples, suggesting distinct viral communities across GIT regions and ruminant species. For instance, RGVC7612 was prevalent across multiple GIT regions in dairy cattle but absent in other species, while RGVC562 was exclusively identified in the large intestine of goats, roe deer, and water deer. The NMDS analysis indicated a clear separation of viral communities by ruminant species (PERMANOVA, *R^2^* = 0.088, *p* < 0.001) and GIT regions (PERMANOVA, *R^2^* = 0.136, *p* < 0.001) (Fig. 2C). Notably, GIT regions had greater variance (31.2 %) in community assembly than ruminant species (22.1 %) (Fig. 2D), highlighting the dominant influence of GIT regions on viral community structure. Alpha-diversity analysis indicated that the stomach and large intestine had the highest Shannon indices and Chao1 richness (Fig. 2E; Table S11), which aligns with rumen being the largest compartment in the ruminant GIT and hosting the highest bacterial populations [89]. Such distinct viral communities among GIT regions could be attributable to multiple factors, such as substrate availabilities and microbial metabolism [6]. The small intestines have bile acids, robust immune activity, and rapid chyme transit, which can inhibit microbial colonization [90], and thus have lower the diversity of viruses (Table S11). These findings underscore that GIT regions, rather than ruminant species, are the primary drivers of viral community diversity in ruminants, aligning well with the distribution patterns of prokaryotic communities within the ruminant GIT [6].

### Virus-host linkages are diverse and widespread in ruminant GIT

To further investigate virus-host linkages, we performed host prediction for all vOTUs against 16,679 MAGs (see Materials and methods; Tables S12-S13). This analysis linked 5954 vOTUs to 4603 putative prokaryotic hosts, generating a total of 15,283 virus-host pairs (Table S14). A substantial proportion of these host-linked vOTUs (n = 2724, 45.8 % of total) possessed multiple host pairings (Fig. S5; Table S15). Among these pairings, 60.4 % had a host range restricted to the same species, genus, or family. A particularly striking example of broad tropism was observed in a subset of 244 viruses, which were predicted to span hosts across multiple phyla. Remarkably, 126 host-linked viruses were predicted to infect both bacteria and archaea, which aligns with prior observations in deep-sea sediment environments [66]. However, virus-host linkages are likely underestimated in our study, as host-linked viruses only accounted for 13.5 % of total vOTUs (Fig. S5; Table S14). Such low proportion of host-linked viruses is consistent with published literature across diverse environments (*e.g.*, 10.4 % in the rumen [29], 14.2 % in deep-sea sediments [66], and 17.9 % in the early-life human gut [91]). The stringent host prediction strategy relied exclusively on a custom ruminant-derived MAG catalog and required zero-mismatch alignment for both CRISPR spacers and tRNA sequences. These two pairing methods achieve high confidence at the expense of low sensitivity, and thus have the inherent limitations of relying on specific genetic signals for virus-host linkage [92,93]. Although these methods capture only a subset of interactions between viruses and hosts, the resulting virus-host dataset establish a high-confidence foundation for analyzing interaction patterns between host-linked viruses and prokaryotic hosts.

Putative prokaryotic hosts spanned 31 bacterial classes and 3 archaeal classes, with the top two bacterial classes being *Clostridia* (n = 7917, 51.8 % of all virus-host pairs) and *Bacteroidia* (n = 4330, 28.3 %) being dominated by the genera *Faecousia* (n = 1110) and *Prevotella* (n = 1066), respectively (Fig. 3; Table S14). *Methanobrevibacter* (including *Methanobrevibacter A/B*, n = 194, 1.27 %), the most abundant genus of methanogens in ruminants [94], is the primary predicted host of archaeal viruses. Surprisingly, the number of viral matches among individual MAGs could vary significantly, ranging from 1 to 108 (Fig. 3), even within the same genus (*e.g.*, *RUG14822*). Shortly, these results indicate prokaryotic hosts are widespread and play roles of reserving viruses, thus can contribute to maintaining the GIT microecology in ruminants.

**Fig. 3:**
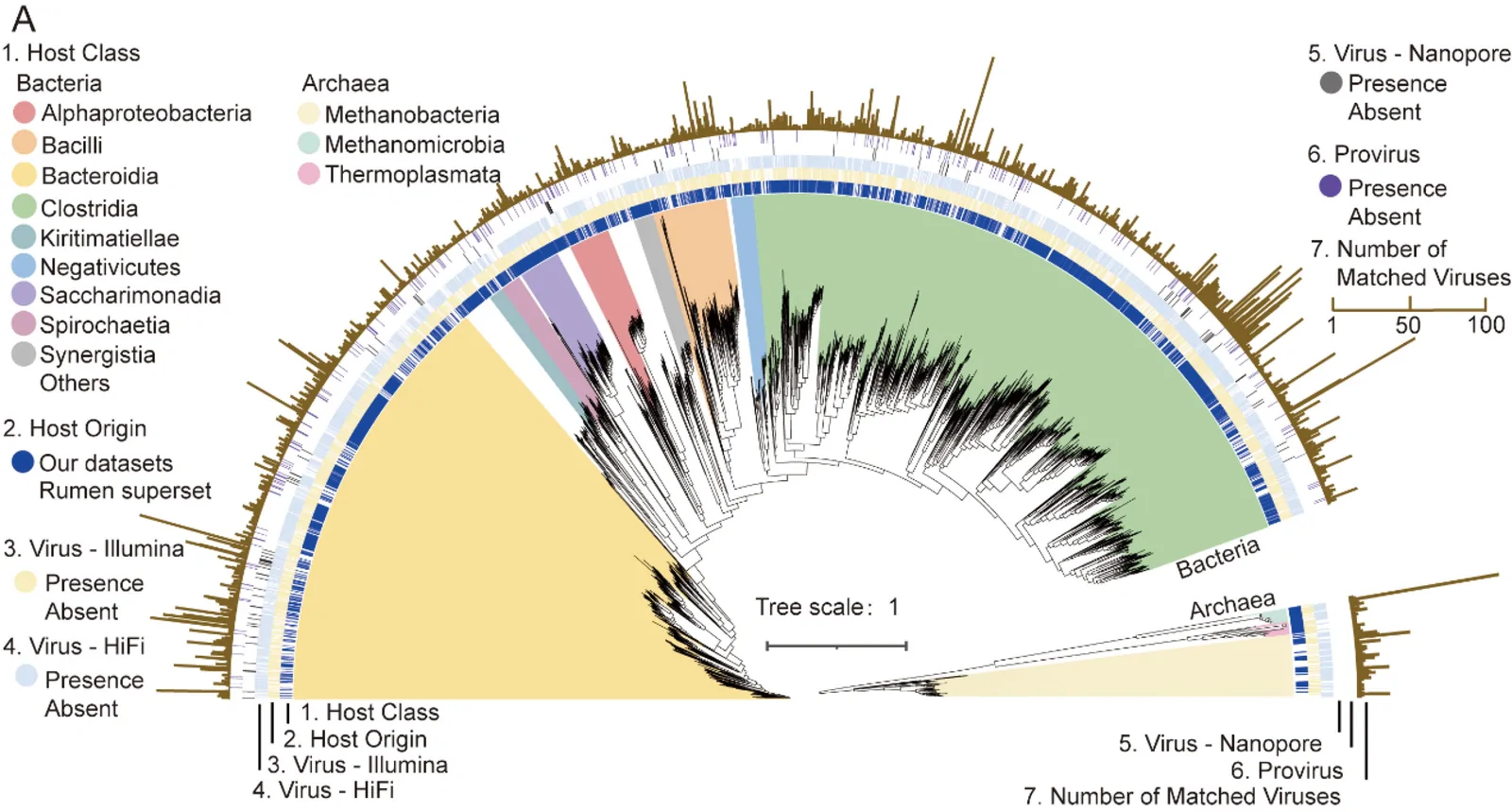
Maximum-likelihood phylogenetic trees of putative prokaryotic MAGs. The concentric rings show: taxonomic class, data origin, presences of linked viruses from Illumina, HiFi, and Nanopore sequencings, presence of linked provirus, and the total number of linked viruses.

### A strong relationship between host-linked virus and prokaryotic hosts contribute to viral spatial heterogeneity in ruminant GIT

Although viruses and their respective hosts exhibited substantial diversity in ruminant GIT, a clear correspondence emerged between the viral composition and that of their putative host (Fig. 4A; Table S16). The class *Bacteroidia* and its predicted viruses had the highest predominance of 54.4 % and 40.8 %, respectively, although the class *Clostridia* had more virus-host linkages. Interestingly, the lineage-specific virus-to-host abundance ratios (VHR) for all taxa exceeded 1, with *Kapabacteria* reaching the highest ratio at 14.7 (Table S16). Such a high VHR value indicates various active states of viral genome replication [66]. Furthermore, a robust positive correlation (*R^2^* = 0.91; *P* < 0.001) was observed between abundances of putative hosts and viruses (Fig. 4B), upholding the close connections of these diverse virus-host linkages among GIT regions.

**Fig. 4:**
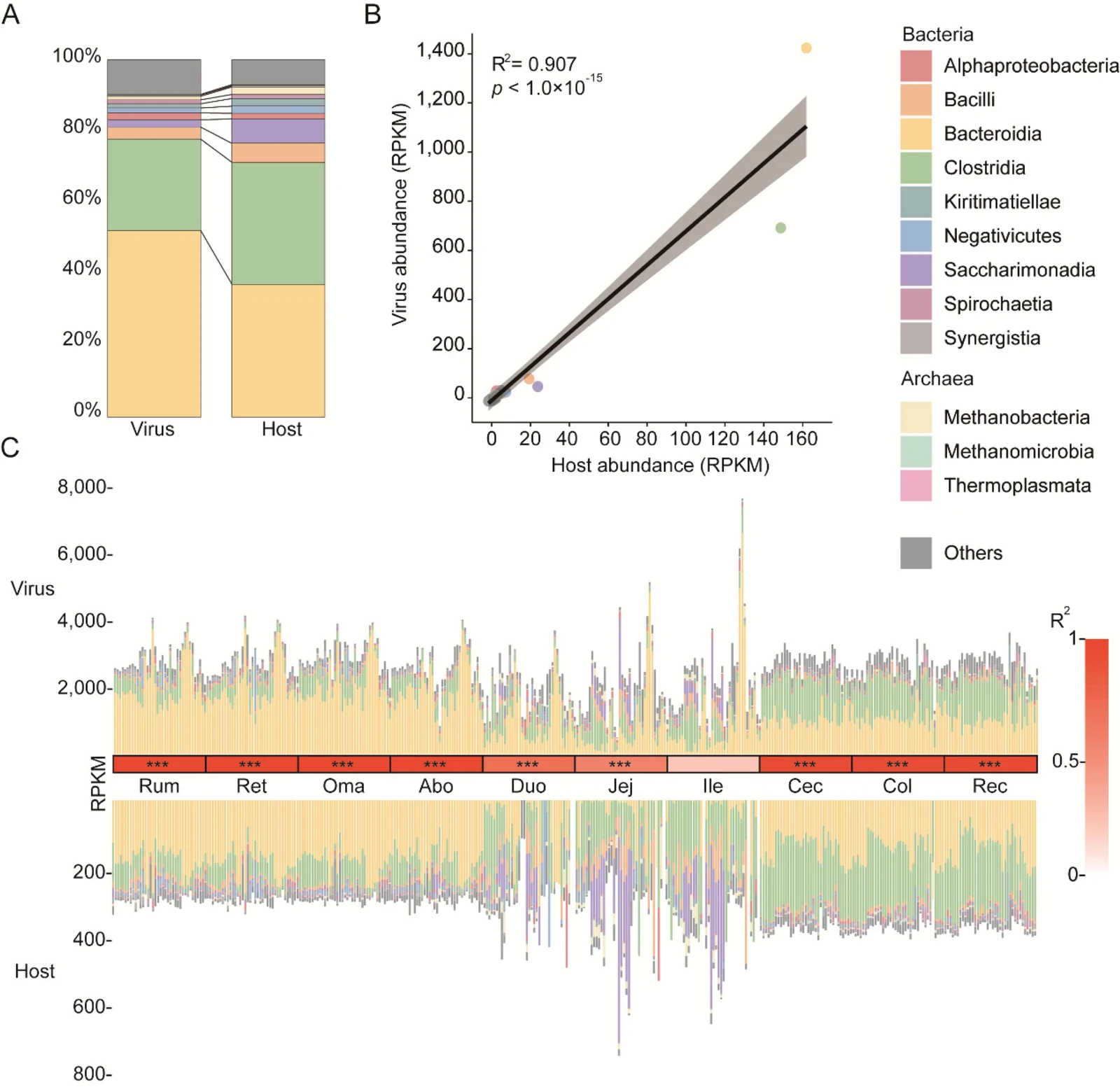
Dynamic correlation between viruses and their putative hosts. A, Relative abundances of vOTUs and their putative hosts grouped by the host class level; B, Significant Pearson correlation between the relative abundances of vOTUs and hosts (calculated by RPKM); C, Pearson correlation between RPKM of vOTUs and their hosts in each gastrointestinal region, with color intensity indicating R^2^ values. Rum, rumen; Ret, reticulum; Oma, omasum; Abo, abomasum; Duo, duodenum; Jej, jejunum; Ile, ileum; Cec, cecum; Col, colon; Rec, rectum. *** *p* < 0.001.

We also detected a distinct and dynamic shift in the viral communities and prokaryotic host communities across the 10 GIT regions (Fig. 4C; Tables S17–18). *Bacteroidia* had the highest abundance in the stomach as virus hosts, followed by the class *Clostridia*, while the opposite was noted in the large intestine. Certain hosts were exclusively present in a specific GIT region; for instance, the class *Synergistia* was virtually absent in the large intestine, whereas *Methanomicrobia* was found solely in the large intestine. Other bacterial and archaeal hosts also exhibited variable abundance (Table S17). Such variations in host abundance were highly correlated with the abundance of the viruses among the GIT regions (*R^2^* ≥ 0.59, *p* < 0.001), except for ileum (Fig. 4C; Table S19). This observation upheld that variation in prokaryotic host communities was closely linked with the spatial heterogeneity of viral communities. Such a robust and interdependent relationship also indicates favorable coexistence between the viral and host communities [95], which exhibits key characteristics of the ‘piggyback-the-winner’ model [24]. Specifically, the positive correlation between host and viral abundances indicates a predominance of temperate strategies, wherein viruses shift from lytic to lysogenic replication as host density increases [96]. This dynamic allows viral persistence without collapsing the host population, thereby facilitating favorable coexistence and maintaining community integrity. The lack of the above correlations in the small intestine is likely attributed to the different intricate microbiome landscapes compared to the stomach and large intestine [97,98]. Collectively, these findings unveil a strong correlation between prokaryotic hosts and host-linked viruses among GIT regions, which may contribute to the wide-varied spatial heterogeneity in viral communities.

### Lysogenic lifestyle is predominant for host-linked viruses with regional variations

As viruses have two lifestyles of lysogenic and lytic features, we then identify provirus and searched for the lysogeny-specific genes to further investigate the metabolic characteristics of the host-linked virus. Approximately 20,050 vOTUs, accounting for 45.6 % of the total, were identified as lysogenic viruses among the 43,981 vOTUs cataloged in the RGVC (Fig. 5A; Table S20). The rest of the host-linked viruses were classified as either lytic (n = 1796) or of unknown lifestyle (n = 22,135). Our proportion of lysogenic viruses (45.6 %) might be an underestimated, as most of the vOTUs in the RGVC correspond to incomplete viral genomes. When using high-quality (>90 % completeness) and complete genomes of viruses, the proportion of lysogenic viruses reached 52.5 % and 59.9 % respectively. Among these lysogenic viruses, 3085 vOTUs were proviruses integrated into host genomes, while 16,965 vOTUs were temperate viruses carrying diverse lysogeny-specific genes (*e.g.*, integrases, excisionases, transposases and recombinases) (Fig. 5B). This functional diversity of lysogeny-specific genes suggests the presence of multiple lysogenic pathways and insertion sites for these viruses [99], enabling viral populations to evade host immunity and adapt to environmental changes [100], [101], [102]. Despite this, integrase genes, particularly those associated with the PF00589 family found in 11,613 vOTUs, emerged as the most common protein families identified (Fig. 5C; Table S21). These findings suggest that approximately half of the viruses in the ruminant GIT are lysogenic, engaging in a symbiotic relationship with their hosts.

**Fig. 5:**
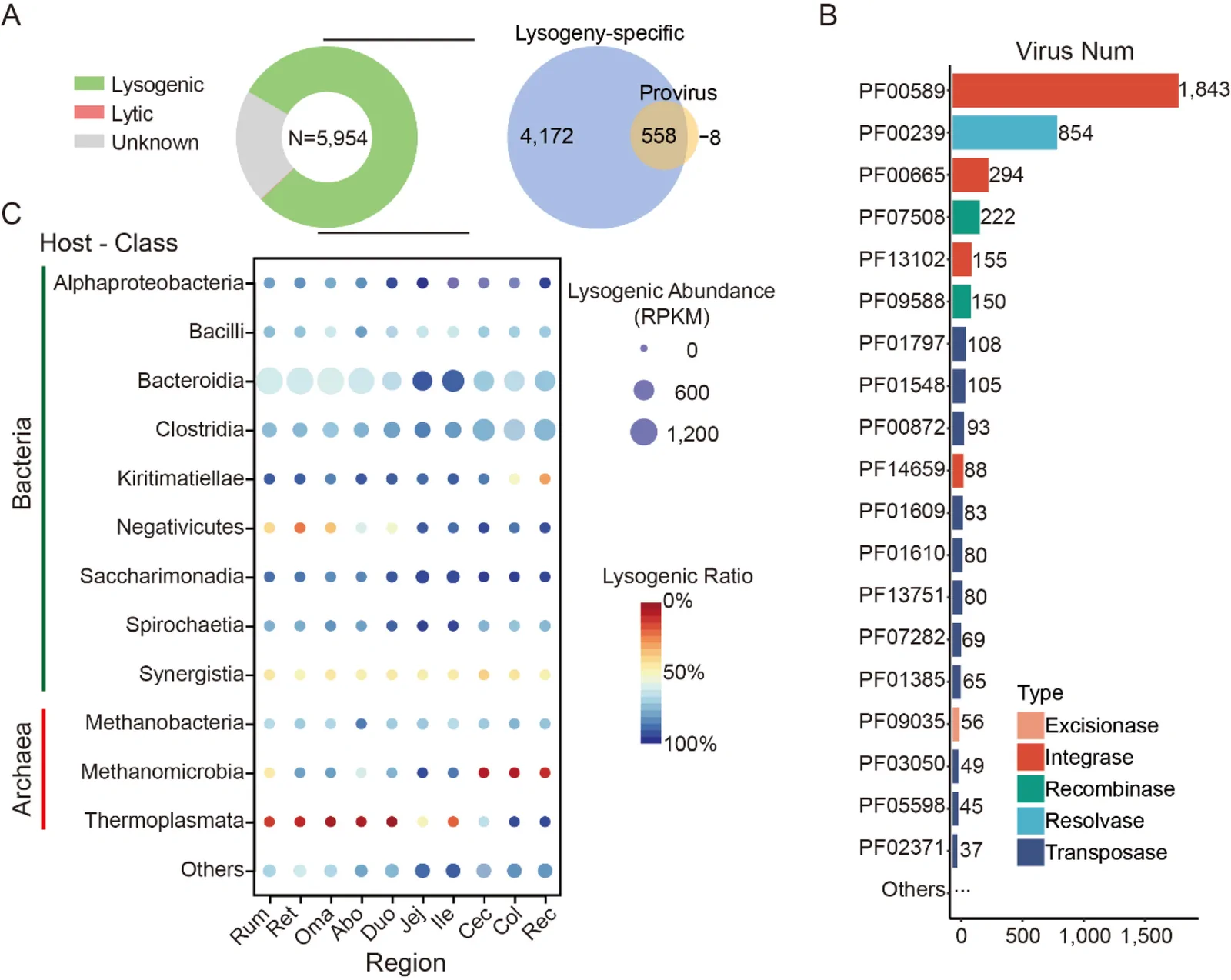
Lifestyles of viruses and inter-regional variations. A, Viral lifestyles of 43,981 vOTUs; lysogenic viruses were predicted based on proviruses and the presence of lysogeny-specific genes; the Venn diagram illustrates the numbers of unique and shared lysogenic viruses between these two approaches; B, Number of viruses with lysogeny-specific genes. Genes are classified by comparison to the Pfam database, and those with the number less than 100 are not shown; C, Comparison of relative abundance ratios of lysogenic host-linked viruses across hosts classes and regions. Rum, rumen; Ret, reticulum; Oma, omasum; Abo, abomasum; Duo, duodenum; Jej, jejunum; Ile, ileum; Cec, cecum; Col, colon; Rec, rectum.

Although the lysogenic lifestyle was dominant in host-linked viruses across all the GIT regions, the lifestyle feature of host-linked viruses varied based on the host classes and specific GIT regions (Fig. 5D). For example, about 50 % of the host-linked viruses infecting the class *Synergistia* were lysogenic, a trend that was consistent across all GIT regions despite significant fluctuations in viral abundance (Fig. 5D; Table S22). However, the proportion of lysogenic viruses exhibited regional variations for *Kiritimatiellae*, *Negativicutes*, *Methanomicrobia,* and *Thermophasmata*. Variations in the lifestyle of these potentially predominant host-linked viruses among the GIT regions could be related to their environmental factors like pH and the ruminant host’s growth status. Lytic viruses are more sensitive to environmental changes than lysogenic ones, which is consistent with the ‘kill-the-winner’ theory [24,103]. Viral lifestyles in the human gut show a similar pattern, with lytic viruses being more abundant while lysogenic viruses being more prevalent [104]. These results indicate that the prevalence of lysogenic lifestyles can contribute to the observed linkage between viruses and prokaryotic hosts, which is critical to maintaining viral survival and diversity in the GIT of ruminants.

### Lysogenic host-linked viruses harbor AMGs encoding putative metabolic functions in prokaryotic hosts

Since lysogenic lifestyle is dominant for host-linked viruses, and considering their potential to modulate host metabolism [18], we specifically searched for AMGs within the genomes of lysogenic host-linked vOTUs. A total of 864 AMGs were detected from 446 lysogenic host-linked vOTUs (Table S23). Among these, 610 genes were classified as Class Ⅰ AMGs (Fig. 6A; Table S24), defined as those encoding enzymes directly involved in KEGG metabolic pathways [43]. Furthermore, 254 AMGs fell into Class II, representing a lower-confidence tier of metabolic genes not directly integrated into core pathways but encoding auxiliary functions such as CAZymes (n = 63) (Table S25) and peptidases (n = 18) (Table S26). The amino acid metabolism (n = 259) emerged as the most prevalent AMG category (Fig. 6B), with enzymes (*e.g.*, homoserine succinyltransferase and arginine deiminase) pointing to a potential role in synthesizing key amino acids (*e.g.*, lysine and arginine), implying that such viral activity may influence host amino acid pool in the gut environment [105]. In nucleotide metabolism pathway (n = 223), the most prevalent AMGs were dUTP pyrophosphatase, ribonucleoside-triphosphate reductase, and DNA (cytosine-5)-methyltransferase 1, which are fundamental to viral replication and aligned with their widespread occurrence as documented in earlier reports [25,32]. Noteworthy, we also identified two valuable AMGs, involved in the carbohydrate and energy metabolism, respectively. The AMG encoding glucose-6-phosphate isomerase (GPI), mediating a pivotal reaction in Embden-Meyerhof pathway, potentially enhancing energy production in the host. In addition, AMGs related to formate-tetrahydrofolate (FHS), a key enzyme through the Wood-Ljungdahl pathway, provided strong evidence for the potential horizontal gene transfer for acetogens in the GIT of ruminants [106]. Collectively, these AMGs point to a potential role in modulating host metabolic processes, providing compelling evidence for a functional linkage between lysogenic virus and their prokaryotic host.

**Fig. 6:**
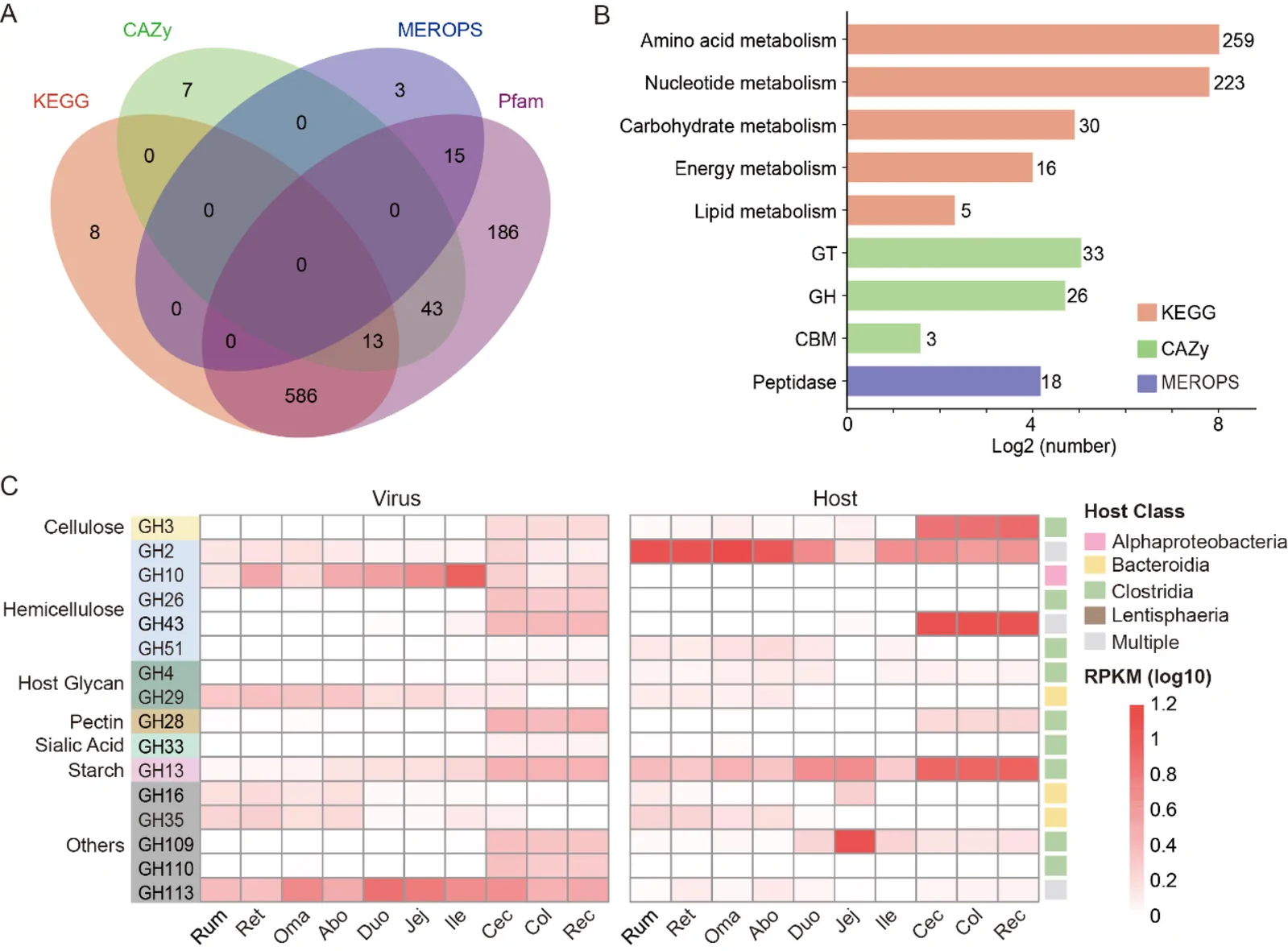
Functional annotation and abundance of selected virus-encoded AMGs. A, Comprehensive annotation of all identified AMGs using the DRAM-v tool, categorized according to four major databases: KEGG, CAZy, MEROPS, and Pfam. B, Counts of AMGs across major functional categories. Bar colors represent the source database (KEGG, CAZy and MEROPS), with exact numbers labeled. C, Spatial co-occurrence of viral GH-encoding AMGs and host GH genes across the ruminant GIT. The heatmap displays the relative abundance (RPKM) of GH from viruses (left) and their hosts (right), annotated via the CAZy database. Putative substrates for each GH family are indicated on the left. The host class is color-coded at the bottom.

The abundance of AMGs also exhibited spatial heterogeneity along the GIT, a pattern particularly evident by GHs. In total, we identified 26 GH-coding AMGs from 21 vOTUs, which were classified into 16 families. These GHs were annotated to enzymes involved in the hydrolysis of polysaccharides abundant in the GIT of ruminants, including fibers, starch, pectin, and host glycans (Fig. 6C), indicating the potential for viruses to enhance host polysaccharide utilization. The abundance of these GH-coding AMGs varied across the GIT regions, partly like the GHs encoded by the host genomes, further corroborating the virus-host linkages distinct to individual GIT regions. In particular, GH3 and GH43 were enriched in the large intestines, whereas GH16 and GH35 were more common in the stomach (Fig. 6C; Table S27). This spatial variation likely reflects a combination of gene transfer events between viruses and their local hosts, coupled with regional selection pressures imposed by distinct dietary substrates available along the GIT [107], driving functional adaptation of both viral and host genomes to their specific ecological niches. Some AMGs, specifically those encoding GH2 and GH10, were found in multiple viruses infecting prokaryotic hosts across various classes, indicating their widespread presence in the GIT of ruminants and thus provide a general advantage for viral spread. These variations in AMG abundance points to generalist and specialist viral strategies, jointly determined by substrate availability and host specificity.

### Genomic context and protein structure alignment of selected AMGs with target function verifies metabolic feature of host-linked viruses

To verify the authenticity and functional reliability of AMGs involved in the carbohydrate and energy metabolism, we further performed a rigorous validation for four selected functions of GH10, GPI and FHS following the standard proposed by Pratama et al. [43]. The genomic context of the AMGs was firstly evaluated based their positions and the annotations of their flanking genes to exclude false positives arising from host contamination [75]. These three AMGs were situated in the central contig regions, beyond the terminal 5 kbp, as indicated by the absence of an 'F' marker in DRAM-v, strengthening the case against host contamination (Fig. 7A). Moreover, these centrally located AMGs were each flanked by a minimum of two hallmark or viral-like genes (*e.g.*, phage integrase, DNA ligase, and transposase) as identified by VirSorter2 [47] and corroborated by the Virus Orthologous Groups Database (VOGDB) [108], thereby substantially increasing confidence in their viral origin. The predicted 3D structures of these four AMGs were queried against reference proteins from the PDB, with the most structurally similar proteins consistently corresponding to their functional annotations. Subsequent quantitative alignment yielded RMSD values between 0.836 Å and 1.691 Å, confirming the high fidelity of the structural models (Fig. 7B). As direct evidence of metabolic reprogramming, the validation of these AMGs not only confirms their viral origin and protein function but also provides robust support for the diverse and complex virus-host linkages. In summary, our results suggest that lysogenic viruses may modulate host metabolic capabilities by providing diverse AMGs, thereby fostering a mutualistic relationship that underscores the dynamic interplay between viruses and their hosts.

**Fig. 7:**
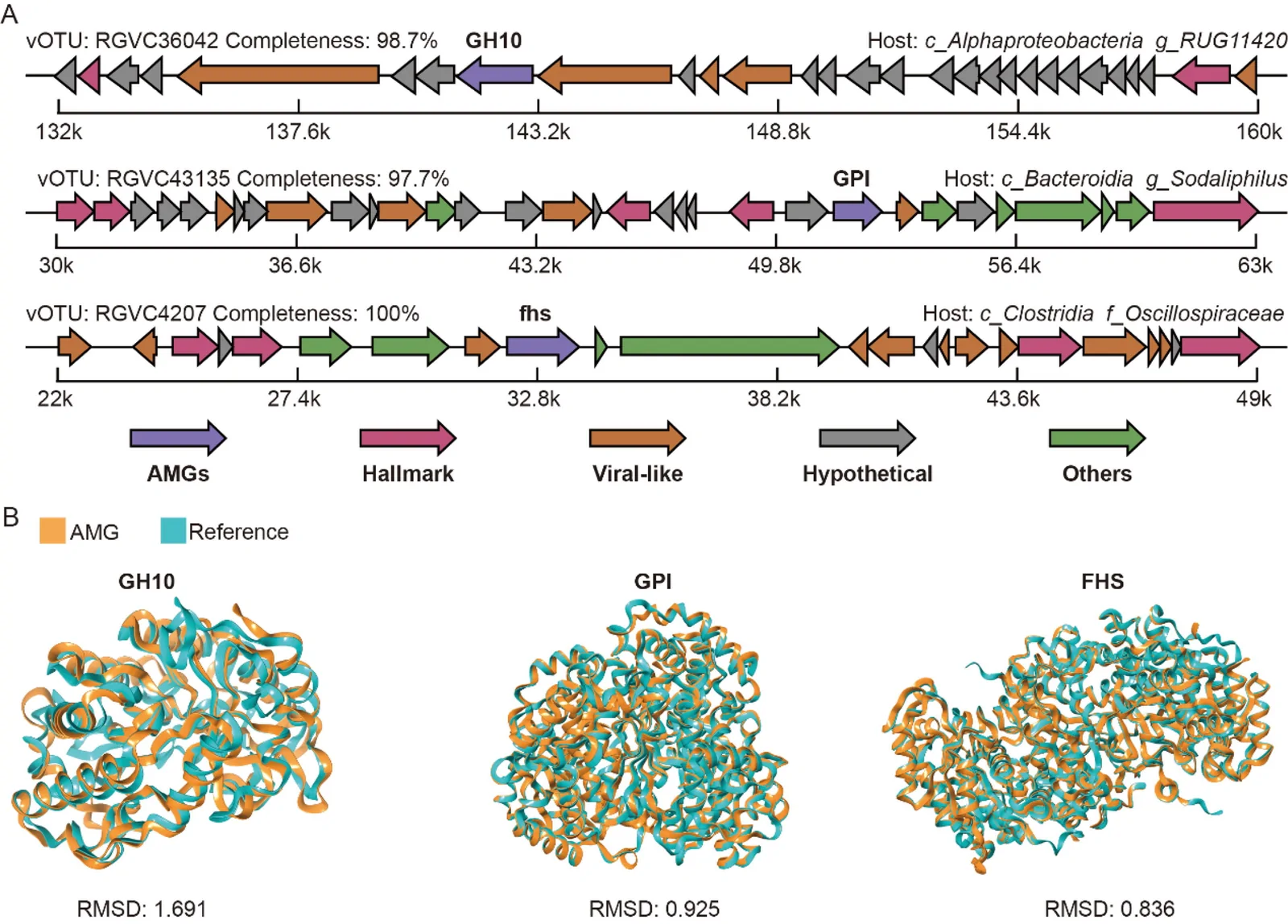
Genomic context and protein structure of selected virus-encoded AMGs. A, Genome map of representative AMG-encoding viruses. Genes are color-coded as follows: purple, the AMG of interest; rose, viral hallmark genes; orange, other viral-like genes; gray, hypothetical proteins; green, non-viral-like genes. Gene direction (forward or reverse strand) is indicated by arrows. Scale bars denote genomic positions. B, Predicted protein structures of four selected AMGs. The 3D structure of each AMG was predicted using AlphaFold3 and structurally aligned to its closest template in the Protein Data Bank (PDB) using the Schrödinger software suite. The Root-Mean-Square Deviation (RMSD) value for each structural alignment is provided. GPI, glucose-6-phosphate isomerase; fhs, formate--tetrahydrofolate ligase.

## Conclusions

Ruminant Gastrointestinal Virome Catalog (RGVC) were reconstructed with 43,981 vOTUs, and provides the most comprehensive characterization to date of viral diversity, composition, and ecological function across 10 GIT regions of seven ruminant species. The distinct heterogeneity of viral diversity and community composition is primarily structured by GIT region rather than host species, with the rumen serving as the principal reservoir of viral richness. Further host-virus analysis uncovered 15,283 high-confidence virus–host pairings with 4603 putative prokaryotic hosts for 5954 vOTUs, highlighting the extensive infection networks between viruses and prokaryotic taxa. The robust correlations observed between abundances of prokaryotic hosts and host-linked viruses across GIT regions suggest that variations in prokaryotic host communities contribute to the assembly of viral communities. Nearly half of host-linked vOTUs exhibit a lysogenic lifestyle, demonstrating that temperate viruses play a central role in maintaining community stability and mediating long-term host–virus coexistence. Functional annotation of lysogenic host-linked viruses identified 864 AMGs, including genes encoding glycoside hydrolases and enzymes related to carbohydrate, amino acid, and hydrogen metabolism of host prokaryote. These metabolic feature of host-linked viruses were further verified by genomic context of selected AMGs with target function.

Collectively, the RGVC establishes a foundational reference for exploring ruminant viral ecology, unveiling the hidden virosphere that shapes spatial heterogeneity of viral communities within the ruminant GIT. These findings provide a mechanistic basis for future interventions aimed at modulating rumen function, such as improving feed efficiency or reducing methane emissions through viral-host interactions. However, translating these insights into applications requires future work to functionally validate the precise mechanisms of AMGs and to elucidate how dynamic factors like diet influence these viral communities. This study represents the first exploration into the spatial heterogeneity of viral communities within the ruminant GIT, providing critical insights that can pave the way for subsequent studies on the intricate interactions between viruses and their hosts, with significant implications for nutrition, health and methane emissions in ruminants.

## Compliance and ethics

All animal experiments complied with the ARRIVE guidelines and carried out the National Research Council's Guide for the Care and Use of Laboratory Animals. All animal-specific procedures were approved and authorized by the Nanjing Agricultural University Institutional Animal Care and Use Committee (No. SYXK-2017–0027), the Ethical Committee of Jilin Agricultural University, and the Chinese Academy of Agricultural Sciences Animal Care and Use Committee (No. ISAPSAEC-2019001).

## Data availability

For Illumina sequencing, raw sequence reads and MAGs are available under the European Nucleotide Archive (ENA) project PRJNA657455 and PRJNA657473, which have been described in our previous study 6. For HiFi and Nanopore sequencing, raw sequence reads and other data supporting the findings of this study are available under the National Genomics Data Center (NGDC) project PRJCA025274. Sequences of 43,981 vOTUs included in RGVC and 659 de-replicated MAGs can be download at figshare (https://figshare.com/projects/RGVC/200877).

## Declaration of competing interest

The authors declare that they have no known competing financial interests or personal relationships that could have appeared to influence the work reported in this paper.
