# Supplementary material for: Spatial heterogeneity of viral communities across the gastrointestinal tracts of ruminants
Source: J Adv Res. 2026 Jan 6;88:31–44. doi: 10.1016/j.jare.2026.01.013 (PMC13669679; doi:10.1016/j.jare.2026.01.013)
Supplement: Supplementary file 1 [file mmc1.docx]

# Supplementary materials and methods

## Samples collection

We selected three rumen samples for DNA sequencing: two (one from a dairy cow and one from a goat) for HiFi sequencing, and one additional dairy cow sample for Nanopore sequencing. Rumen samples were collected 3 h after morning feeding using a stomach tube, with the first 150 mL of rumen contents discarded to avoid saliva contamination [1]. To obtain a representative view of the entire rumen virome, the sample was rapidly homogenized without solid–liquid separation. and transferred into sterile DNase- and RNase-free tubes. These sample tubes were immediately frozen in liquid nitrogen and then stored at -80℃ until DNA extraction.

## Microbial DNA extraction

Microbial DNA was extracted following the protocol of Yu and Morrison [2] and purified with phenol/chloroform/isoamyl alcohol (25:24:1, vol/vol/vol; Solarbio, Shanghai, China) as described by Minas et al. [3]. The DNA pellets were vacuum-dried, washed twice with 70% ethanol, and resuspended in Tris-EDTA buffer (10 mM Tris, 1 mM EDTA, pH 8.0). DNA concentration and purity were assessed using a NanoDrop ND-2000 spectrophotometer (NanoDrop Technologies, Wilmington, DE), and samples were stored at −80°C until further analysis.

## Metagenomic sequencing and quality control

For short-read sequencing, detailed processes have been reported previously [4]. Briefly, individual metagenomic sequencing libraries with an average length of 350 bp were prepared for all samples using the TruSeq DNA PCR-Free Library Preparation Kit (Illumina, USA) according to the manufacturer’s protocol and sequenced on an Illumina NovaSeq platform. Adapters from the Illumina data were first trimmed using Trimmomatic v.0.33 [5]. To minimize environmental DNA contamination, we then aligned the reads against three reference sets using BWA-MEM v0.7.17 [6]: (1) the host genome, including dairy cattle (*Bos Taurus*, GCA_002263795.2), water buffalo (*Bubalus bubalis*, GCA_003121395.1), yak (*Bos mutus*, GCA_000298355.1), goat (*Capra hircus*, GCA_001704415.1), sheep (*Ovis aries*, GCA_002742125.1), roe deer (*Capreolus pygargus*, GCA_000751575.1) and water deer (*Hydropotes inermis*, GCA_006459105.1); (2) plant genomes representative of the animals’ diets, including sorghum (*Sorghum bicolor*, GCA_000003195.3), wheat (*Triticum aestivum*, GCA_002220415.3), robur (*Quercus robur*, GCA_900291515.1), sweet potato (*Ipomoea batatas*, GCA_002525835.2), medicago (*Medicago truncatula*, GCA_000219495.2), rice (*Oryza sativa*, GCF_000005425.2), barley (*Hordeum vulgare*, GCA_900075435.2), maize (*Zea mays*, GCA_003185045.1 and GCA_000005005.6), soybean (*Glycine max*, GCA_000004515.4), and ryegrass (*Lolium perenne*, GCA_001735685.1); and (3) the human genome (*Homo sapiens*, GCA_000001405.28).

For HiFi sequencing, about 5 μg of DNA was used to prepare a SMRTbell library with the PacBio SMRTbell prep kit 3.0 (Pacific Biosciences) according to the manufacturer’s recommendations. Damaged double-stranded DNA in the initial DNA sample was repaired using the New England BioLabs PreCR Repair Mix Kit before library preparation [7]. Then, the repaired DNA was size-selected by using the BluePippin system (Sage Science) to obtain molecules larger than 3 kb. SMRTbell libraries were prepared for each sample using the PacBio SMRTbell Preprocessing Kit 3.0 and then sequenced by v3 chemistry on a PacBio Sequel Iie instrument using SMRT 8M cells. Finally, HiFi reads (45.8 Gbp from dairy cattle and 31.8 Gbp from goat) were generated by the ‘ccs’ module in the SMRT Link v10.0 (Pacific Biosciences) with parameters ‘-min-length 100 -min-passes 3 -min-rq 0.99’.

For Nanopore sequencing, DNA samples were quantified using a Qubit® 3.0 Fluorometer (Invitrogen, USA). Long DNA fragments were size-selected using the PippinHT system (Sage Science, USA), followed by end-repair and adapter ligation with the SQK-LSK114 kit (Oxford Nanopore Technologies, UK). The final library concentration was measured again using the Qubit® 3.0 Fluorometer before loading onto a primed Nanopore PromethION flow cell for sequencing. Raw sequencing signals were base-called and demultiplexed using Guppy v0.1.10 [8], yielding 194 Gbp of Nanopore reads. Residual adapters were then trimmed using Porechop v0.2.4 (https://github. com/rrwick/Porechop).

## Metagenomic binning and genome quality assessment

For the metagenomic binning step, contigs larger than 1500 bp from each assembly (generated from HiFi and Nanopore sequencing) were independently processed using metaWRAP v1.3.2 [9] with parameters: ‘--metabat2 --maxbin2 --concoct --universal’. Then the MAGs generated from the different methods were filtered, dereplicated, and aggregated using DASTool v1.1.6 [10] with default parameters. Evaluation using CheckM2 v1.0.1 [11] identified 659 high-quality MAGs (completeness ≥ 50% and a contamination rate ≤ 10%).

## References

1.	Wang, M., et al., *Sampling procedure for the measurement of dissolved hydrogen and volatile fatty acids in the rumen of dairy cows.* Journal of Animal Science, 2016. **94**(3): p. 1159-1169.

2.	Yu, Z. and M. Morrison, *Improved extraction of PCR-quality community DNA from digesta and fecal samples.* Biotechniques, 2004. **36**(5): p. 808-12.

3.	Minas, K., et al., *Optimization of a high-throughput CTAB-based protocol for the extraction of qPCR-grade DNA from rumen fluid, plant and bacterial pure cultures.* FEMS Microbiology Letters, 2011. **325**(2): p. 162-169.

4.	Xie, F., et al., *An integrated gene catalog and over 10,000 metagenome-assembled genomes from the gastrointestinal microbiome of ruminants.* Microbiome, 2021. **9**(1): p. 137.

5.	Bolger, A.M., M. Lohse, and B. Usadel, *Trimmomatic: a flexible trimmer for Illumina sequence data.* Bioinformatics, 2014. **30**(15): p. 2114-20.

6.	Li, H. and R. Durbin, *Fast and accurate short read alignment with Burrows-Wheeler transform.* Bioinformatics, 2009. **25**(14): p. 1754-60.

7.	Tao, Y., et al., *Improved Assembly of Metagenome-Assembled Genomes and Viruses in Tibetan Saline Lake Sediment by HiFi Metagenomic Sequencing.* Microbiology Spectrum, 2023. **11**(1): p. e03328-22.

8.	Wick, R.R., L.M. Judd, and K.E. Holt, *Performance of neural network basecalling tools for Oxford Nanopore sequencing.* Genome Biology, 2019. **20**(1): p. 129.

9.	Uritskiy, G.V., J. DiRuggiero, and J. Taylor, *MetaWRAP—a flexible pipeline for genome-resolved metagenomic data analysis.* Microbiome, 2018. **6**(1).

10.	Sieber, C.M.K., et al., *Recovery of genomes from metagenomes via a dereplication, aggregation and scoring strategy.* Nat Microbiol, 2018. **3**(7): p. 836-843.

11.	Chklovski, A., et al., *CheckM2: a rapid, scalable and accurate tool for assessing microbial genome quality using machine learning.* Nature methods, 2023. **20**(8): p. 1203-1212.
