# Supplementary material for: Spatial heterogeneity of viral communities across the gastrointestinal tracts of ruminants
Source: J Adv Res. 2026 Jan 6;88:31–44. doi: 10.1016/j.jare.2026.01.013 (PMC13669679; doi:10.1016/j.jare.2026.01.013)
Supplement: Supplementary file 2 [file mmc2.docx]

**Fig S1. Number of viral contigs identified by different detection methods across three sequencing datasets.**

A, Bar plot showing the number of putative viral contigs identified by each of the five tools (IMG/VR, VirFinder, DeepVirFinder, VirSorter2, and VIBRANT) across the Illumina, Nanopore and HiFi sequencing datasets. B, The UpSet plot illustrating the intersections and unique contributions of the five detection methods. The vertical bars represent the size of contig sets that are either unique to a single tool or shared by specific combinations of tools (as indicated by the connected dots below).

**Fig S2. Ruminant species and gastrointestinal region origins of 43,981 vOTUs.** A, Proportions of vOTUs identified from the ruminant species; B, Proportions of vOTUs identified at each gastrointestinal region. Colors represent distinct species or regions, and percentages indicate the proportion of vOTUs relative to the total dataset.

**Fig S3. Comparison of the length of vOTUs sequences obtained from three sequencing datasets**. The violin plot represents the distribution of lengths of vOTUs sequence from HiFi, Illumina, and Nanopore sequencing methodologies, ranging from 5 kbp to 1000 kbp. *** *p*< 0.001

**Fig S4.** **Overview of the process of vOTUs annotation and analysis.** geNomad was used for the taxonomic annotation of 4,3981 vOTUs, together with 685 vOTUs without realm classification. All vOTUs were predicted hosts using three matching methods (CRISPR, tRNA and homology) with the self-constructed MAGs library, resulting in the identification of 4,603 known hosts and 5,954 host-linkage viruses. Lysogenic viruses were detected through signal genes or proviruses from all vOTUs, and lysogenic host-linked viruses were subjected to functional annotation processes, including sequence confirmation and trimming, data preparation, annotation, and verification.

**Fig S5.** **Overview of the matching process between vOTUs and host MAGs.** About 5,954 out of 4,3981 vOTUs were matched with host MAGs (A), in which 2,724 vOTUs had multiple matches (B). C, the range of host range at species, genus, family, order, class, phylum, domain and kingdom levels.
